# Prolonged increased neutrophil-to-lymphocyte ratio is associated with mortality after successful revascularization for treatment of acute ischemic stroke

**DOI:** 10.1186/s12883-022-02847-3

**Published:** 2022-08-31

**Authors:** Song Li, Linghong Hu, Jian Wang, Feihui Zou, Bin Han, Yougang Wang, Kefeng Liu

**Affiliations:** Department of Neurosurgery, Changzhou Second People’s Hospital Affiliated to Nanjing Medical University, No.29, Xinglong Lane, Changzhou, 213004 Jiangsu China

**Keywords:** Neutrophil-to-lymphocyte ratio, Endovascular treatment, Anterior circulation large vessel occlusion stroke; acute ischemic stroke; follow-up, Mortality

## Abstract

**Background:**

To determine the association between dynamic neutrophil-to-lymphocyte ratio (NLR) during hospitalization and mortality 1 month after ischemia reperfusion in patients undergoing endovascular treatment (EVT) with successful revascularization for acute large vessel occlusion stroke.

**Methods:**

This retrospective study included patients who had undergone successful EVT. Information was collected regarding patients’ clinical characteristics, imaging data, and mortality at 1 month. Univariate and multivariate logistic regression models were applied to assess the association between NLR and mortality. We used a generalized additive model and a generalized additive mixed model to compare trends in NLR over time between survivors and nonsurvivors.

**Results:**

A total of 237 patients were included. During the 1-month follow-up, 42 of these patients (17.7%) died. The multivariate analysis demonstrated that NLR obtained within 12 to 24 hours (odds ratio [OR] = 1.18; 95% confidence interval [CI]: 1.04, 1.33; *P* = 0.008), 24 to 48 hours (OR = 1.16; 95% CI: 1.01, 1.35; *P* = 0.044), and 48 to 72 hours (OR = 1.23; 95% CI: 1.03, 1.47; *P* = 0.021) after EVT were independently associated with mortality at 1 month. In addition, there was a trend for NLR to decrease gradually over time for both survivors and nonsurvivors; however, NLR in survivors decreased by an average of 0.29 daily than in nonsurvivors.

**Conclusions:**

Increased NLR in the early period after EVT was associated with an increased risk of mortality, and a continued trend toward higher NLR over time was also linked with a higher mortality risk.

## Background

Endovascular treatment (EVT) has become the gold standard for the treatment of large vessel occlusion in patients with acute ischemic stroke [1]; however, patients undergoing this therapy are still at high risk of a poor prognosis, including mortality as high as 15.3% [2]. The inflammatory mechanism of stroke has been widely researched [3]. A systemic inflammatory response is rapidly induced shortly after ischemia reperfusion injury, and neutrophils in the blood enter ischemic or infarcted tissue through the injured blood-brain barrier and transmit inflammatory factors [4, 5]. Through a variety of mechanisms, inflammatory mediators such as oxygen free radicals and matrix metalloproteinase-9 cause damage to brain cells [6–8]. However, the increased concentration of neutrophils in the peripheral blood is accompanied by a continuous decrease in lymphocytes because of the engagement of T cells and platelets through P-selectin [9]. The activated platelets can prevent bleeding transformation and play a role in hemostasis [10].

Previous studies have shown that the neutrophil-to-lymphocyte ratio (NLR) in the early poststroke period may reflect the state of inflammation and which plays an important role in ischemia-reperfusion injury [11–15], thus NLR is served as an inexpensive and reliable biological indicator [16–19]. However, the correlation between NLR and death after ischemia reperfusion is still controversial [20, 21], and the relationship between dynamic NLR during hospitalization and death in stroke patients with successful revascularization is still unclear.

To address these uncertainties, we collected data on stroke patients undergone successful revascularization for anterior circulation large vessel occlusion stroke, and sought to assess the potential relationship between dynamic NLR during hospitalization and mortality at 1 month, in the hope that a proven correlation would help physicians make treatment decisions.

## Methods

### Patients and population

This case series was approved by the Ethics Committees of Changzhou Second People’s Hospital Affiliated to Nanjing Medical University (Approval No: [2021] YLJSC020). Because this study involved an observational retrospective cohort, written consent from patients was not required.

Consecutive patients aged ≥18 years who underwent EVT for large vessel occlusion stroke at Changzhou Second People’s Hospital Affiliated to Nanjing Medical University between May 2017 and December 2021 were considered for study inclusion. Eligible patients were those who had experienced an anterior circulation large vessel occlusion stroke, confirmed by head computed tomography (CT) angiography or digital subtraction angiography (DSA); those with a modified Rankin Scale score ≤ 1; those with a National Institutes of Health Stroke Scale (NIHSS) score > 5 at admission; and those in whom treatment was initiated within 12 hours after stroke onset .

**Fig. 1:**
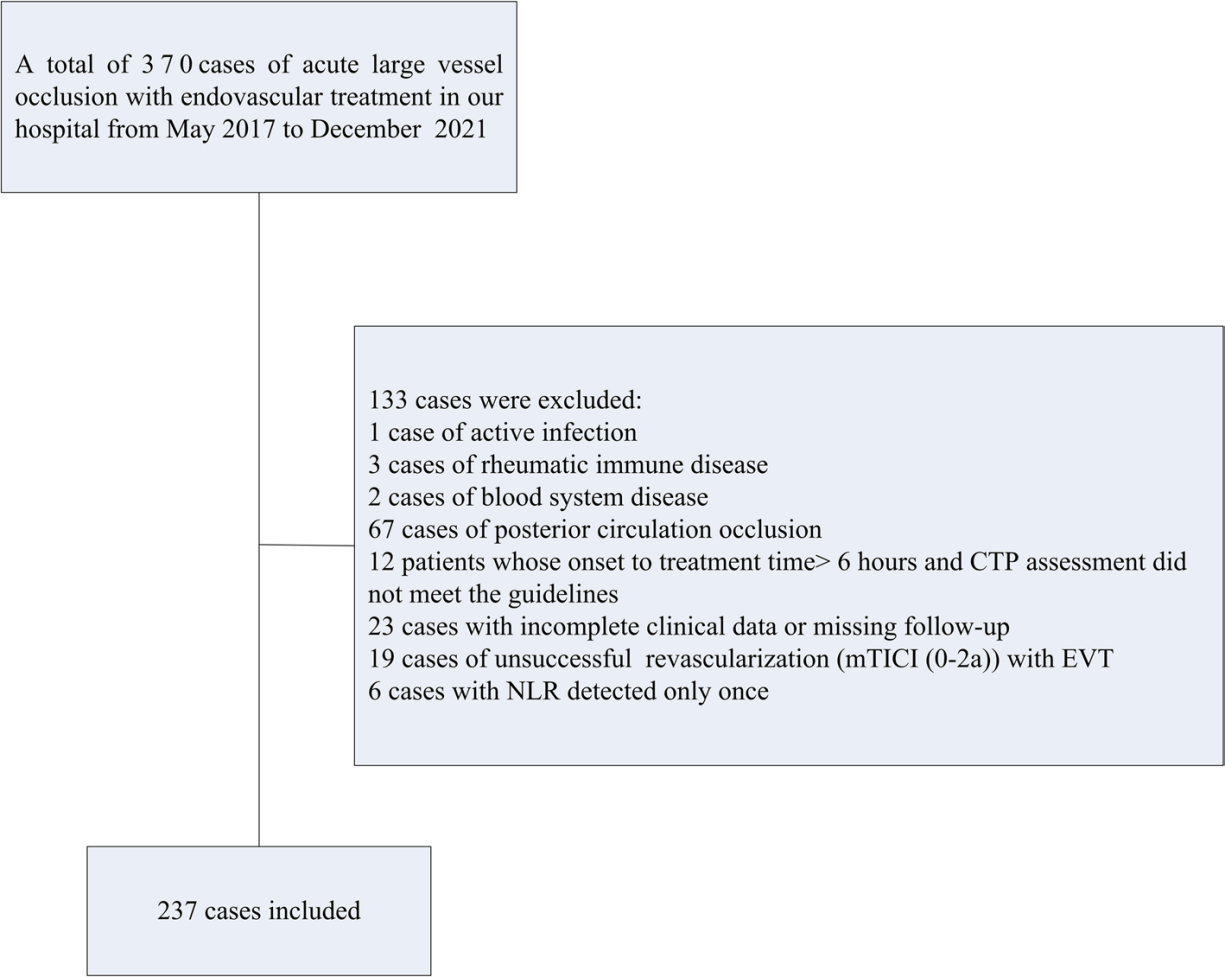
Flow chart of patient selection. CTP, computed tomography perfusion; EVT, endovascular treatment; mTICI, modified Thrombolysis in Cerebral Infarction; NLR, neutrophil-to-lymphocyte ratio

Patients were excluded from the study if > 6 hours after stroke onset and CT perfusion evaluation did not meet established criteria guidelines [1, 22]; if they had an active infection within 3 days before the onset of stroke, confirmed by clinical symptoms, elevated leukocyte levels, or elevated C-reactive protein levels; if they had a history of rheumatic immune disease; if they had incomplete clinical data (eg, missing follow-up, only 1 documented leukocyte/lymphocyte value); or if they had undergone unsuccessful EVT revascularization (ie, modified Thrombolysis in Cerebral Infarction score of 0-2a). Once these criteria were applied, 237 patients were eligible for study inclusion (Fig. 1).

### Data and outcome analysis

We collected information regarding patient demographics, clinical history, past medical history and history of drug use, imaging data, laboratory results, NIHSS score, Alberta Stroke Program Early CT Score (ASPECTS), complications during hospitalization，medication during hospitalization and mortality at 1 month after EVT. DSA was used to assess collateral compensation; grades 3 and 4 were defined as favorable collateral compensation based on guidelines from the American Society of Interventional and Therapeutic Neuroradiology/Society of Interventional Radiology [23].symptomatic intracranial hemorrhage was defined as a hemorrhage volume on CT exceeding 30% of the infarct volume and an increase in the NIHSS score of 4 points within 72 hours.

All leukocyte and lymphocyte counts obtained during hospitalization were collected, and NLR for each sample were calculated by dividing the neutrophil count by the lymphocyte count. NLR were categorized into values obtained at admission, immediately after EVT, within 12 to 24 hours after EVT, within 24 to 48 hours after EVT, and within 48 to 72 hours after EVT.

Mortality at 1 month after EVT was recorded by clinicians either in the hospital or via telephone consultation after the patient had been discharged. All clinical and imaging data were assessed by 2 experienced cerebrovascular physicians who were not familiar with the clinical information and the imaging data. In case of disagreements, a third senior cerebrovascular physician was consulted.

### Statistical analysis

For this analysis, we compared dynamic NLR during hospitalization in patients who were alive and those who had died at 1 month after EVT. Continuous variables were described as mean ± standard deviation or as median (interquartile range [IQR]). Categorical variables were described as rate and composition ratio. Baseline characteristics were analyzed using a *t* test or a Kruskal–Wallis rank-sum test for continuous variables and a chi-square test for categorical variables. Univariate and multivariate logistic regression models were applied to evaluate the association between NLR at different time points and mortality at 1 month after successful revascularization, with adjustments made for multiple confounders in multivariate models. For the multivariate analysis, the covariates were included as potential confounders in the final models if they changed the estimates of NLR on mortality at 1 month by more than 10% or were significantly associated with mortality. One such confounder was the occurrence of pneumonia during hospitalization, as poststroke infections such as pneumonia are common, and the presence of infection will affect the NLR [24–27].

The smoothing plots made by generalized additive model were used to assess NLR and mortality at different points after successful revascularization. The difference in NLR between survivors and nonsurvivors at 1 month after EVT was derived from a generalized additive mixed model. The model is used to analyze repeated measurement data, especially when some data are missing and the time interval for repeated measurements is irregular [28, 29].

All analyses were performed using the R package (http://www.R-project.org; The R Foundation) and EmpowerStats (https://www.empowerstats.com; X&Y Solutions, Inc). A *P* value < 0.05 (two-sided) was considered statistically significant.

## Results

### Baseline characteristics

Of the 370 patients who were treated with EVT during the study period, 237 (64.1%) were included in the analysis (Fig. 1). The baseline characteristics of study patients are shown in Table 1. The study population included 147 men (62.0%), and the median age of patients was 70.0 years (IQR, 63.0–76.0). The median baseline NIHSS score was 15 points (IQR, 10.0–20.0) and the median ASPECTS was 8.0 (IQR, 7.0–9.0). Nineteen (8.0%) patients had symptomatic intracranial hemorrhage. There was not a statistically significant difference in mortality (*P* = 0.088) and symptomatic intracerebral hemorrhage (*P* = 0.400) among the categorical variable (tertile) groups of NLR.

**Table 1:** The characteristics of study participants

Characteristic	Total (*N* = 237)	NLR			*P* value
		Low (*n* = 79)	Middle (*n* = 79)	High (*n* = 79)	
Median age, y (IQR)	70.0 (63.0–76.0)	70.0 (63.5–76.0)	70.0 (58.5–76.0)	72.0 (64.0–77.0)	0.646
Men, n (%)	147 (62.0)	48 (60.8)	52 (65.8)	47 (59.5)	0.686
Medical history, n (%)					
Hypertension	157 (66.2)	52 (65.8)	57 (72.1)	48 (60.8)	0.316
Diabetes	34 (14.4)	11 (13.9)	10 (12.7)	13 (16.5)	0.786
Atrial fibrillation	117 (49.4)	41 (51.9)	43 (54.4)	33 (41.8)	0.242
Coronary heart disease	27 (11.4)	8 (10.1)	12 (15.2)	7 (8.9)	0.416
Stroke	40 (16.9)	18 (22.8)	8 (10.1)	14 (17.7)	0.102
Chronic kidney disease	5 (2.1)	1 (1.3)	2 (2.5)	2 (2.5)	1.000
Carotid artery disease	8 (3.4)	2 (2.5)	2 (2.5)	4 (5.1)	0.736
Current antiplatelet therapy	22 (9.3)	10 (12.7)	4 (5.1)	8 (10.1)	0.246
Current anticoagulation therapy	16 (6.8)	6 (7.6)	7 (8.9)	3 (3.8)	0.418
Clinical characteristics					
Median baseline NIHSS score (IQR)	15.0 (10.0–20.0)	12.0 (10.0–17.0)	14.0 (10.0–20.0)	16.0 (12.0–20.0)	0.028
Median ASPECTS (IQR)	8.0 (7.0–9.0)	8.0 (8.0–9.0)	8.0 (7.0–9.0)	8.0 (7.0–9.0)	0.027
Mean systolic blood pressure at admission ± SD, mmHg	151.1 ± 24.4	150.8 ± 27.4	152.9 ± 24.0	149.7 ± 21.9	0.519
Mean diastolic blood pressure at admission ± SD, mmHg	87.8 ± 14.5	87.0 ± 17.1	89.1 ± 12.1	87.3 ± 13.9	0.494
Mean glucose at admission ± SD, mmol/l	7.8 ± 2.9	7.3 ± 1.9	7.6 ± 2.5	8.3 ± 3.8	0.195
Cause of stroke, n (%)					0.818
Cardioembolic	141 (59.5)	44 (55.7)	48 (60.8)	49 (62.0)	
Intracranial atherosclerosis	54 (22.8)	20 (25.3)	18 (22.8)	16 (20.3)	
Other	42 (17.7)	15 (19.0)	13 (16.5)	14 (17.7)	
Location of occlusion, n (%)					0.760
Internal carotid artery	92 (38.8)	30 (38.0)	34 (43.0)	28 (35.4)	
Middle cerebral artery M1 segment	103 (43.5)	32 (40.5)	34 (43.0)	37 (46.9)	
Middle cerebral artery M2 segment	35 (14.8)	13 (16.5)	10 (12.7)	12 (15.2)	
Anterior cerebral artery A1 segment	7 (3.0)	4 (5.1)	1 (1.3)	2 (2.5)	
Bridging intravenous thrombolysis, n (%)	24 (10.1)	9 (11.4)	4 (5.1)	11 (14.0)	
Collateral circulation, n (%)					0.111
ASITN/SIR 0–2	189 (79.8)	57 (72.2)	65 (82.3)	67 (84.8)	
ASITN/SIR 3–4	48 (20.3)	22 (27.9)	14 (17.7)	12 (15.2)	
Complications during hospitalization, n (%)					
Pneumonia	98 (41.4)	29 (36.7)	36 (45.6)	33 (41.8)	0.525
Urinary tract infection	5 (2.1)	1 (1.3)	2 (2.5)	2 (2.5)	1.000
Sepsis	6 (2.5)	1 (1.3)	3 (3.8)	2 (2.5)	0.873
Any intracerebral hemorrhage	102 (43.0)	27 (34.2)	29 (36.7)	46 (58.2)	0.004
Symptomatic intracerebral hemorrhage	19 (8.0)	5 (6.3)	5 (6.3)	9 (11.4)	0.400
Treatment during hospitalization,n (%)					
Anticoagulation	64 (27.0)	24 (30.4)	20 (25.3)	20 (25.3)	0.710
Dialysis	1 (0.4)	0 (0.0)	0 (0.0)	1 (1.3)	1.000
Invasive mechanical ventilation	51 (21.5)	12 (15.2)	16 (20.3)	23 (29.1)	0.098
Vasopressors	40 (16.9)	9 (11.4)	11 (13.9)	20 (25.3)	0.045
Mortality at 1 moth, n(%)	42 (17.7)	10 (12.7)	12 (15.2)	20 (25.3)	0.088

*NLR* Neutrophil-to-lymphocyte ratio, *ASITN* American Society of Interventional and Therapeutic Neuroradiology, *SIR* Society of Interventional Radiology, *ASPECTS* Alberta Stroke Program Early CT Score, *IQR* Interquartile range, *SD* Standard deviation, *NIHSS* National Institute of Health Stroke Scale

The sample included 195 patients who were alive at 1 month after EVT (82.3%) and 42 patients who had died at 1 month after EVT (17.7%). Among nonsurvivors, the cause of death was cerebral hemorrhage in 13 patients (31.0%), malignant brain swelling in 8 patients (19.0%), respiratory failure in 12patients (28.6%), heart failure in 5 patients (11.9%), septic shock in 2 patients (4.8%), and “other cause” in 2 patient (4.8%). Among them, 21 cases (50%) died of cerebral causes and 21 cases (50%) died of extra-cerebral causes. The median NLR of the two groups was 6.69 (2.40–7.88) and 3.95 (2.62–5.48) respectively, with no significant difference between them (*P* = 0.489). In addition, 30 cases (71.4%) died in hospital.

### Clinical outcomes

Univariate analysis demonstrated that age (*P* = 0.003), baseline NIHSS score (*P* < 0.001), history of diabetes (*P* = 0.001), history of chronic kidney disease (*P* = 0.031), glucose at admission(*P* = 0.017), bridging intravenous thrombolysis (*P* = 0.040), cardioembolism (*P* = 0.040), internal carotid artery occlusion (*P* = 0.049), Pneumonia during hospitalization (*P* = 0.024), anticoagulation during hospitalization (*P* = 0.004), use of invasive mechanical ventilation and vasopressors during hospitalization (*p* < 0.001), sepsis during hospitalization (*P* = 0.001), any intracerebral hemorrhage and symptomatic intracerebral hemorrhage during hospitalization (*p* < 0.001), NLR within 12 to 24 hours after EVT (*p* < 0.001), NLR within 24 to 48 hours after EVT (*P* = 0.002), and NLR within 48 to 72 hours after EVT (*p* < 0.001) were associated with mortality at 1 month (Table 2).

**Table 2:** Univariate analysis of mortality at 1 month after EVT

Variable	Value^**a**^	Mortality at 1 mo	
		OR (95% CI)	***P*** value
Sex			0.287
Male	147 (62.03)	Reference	
Female	90 (37.97)	1.44 (0.74, 2.83)	
Age, y	67.91 ± 11.97	1.06 (1.02, 1.10)	0.003
History of hypertension	157 (66.24)	1.54 (0.73, 3.26)	0.256
History of diabetes	34 (14.35)	3.71 (1.68, 8.23)	0.001
History of atrial fibrillation	117 (49.37)	1.65 (0.84, 3.24)	0.149
History of coronary heart disease	27 (11.39)	0.79 (0.26, 2.41)	0.675
History of stroke	40 (16.88)	0.62 (0.23, 1.68)	0.347
History of chronic kidney disease	5 (2.11)	7.42 (1.20, 45.90)	0.031
History of carotid artery disease	8 (3.38)	2.92 (0.67, 12.74)	0.153
Current antiplatelet therapy	22 (9.28)	1.41 (0.49, 4.08)	0.520
Current anticoagulation therapy	16 (6.75)	1.08 (0.29, 3.96)	0.911
Systolic BP at admission, mmHg	151.09 ± 24.43	1.00 (0.99, 1.02)	0.807
Diastolic BP at admission, mmHg	87.78 ± 14.46	0.99 (0.97, 1.02)	0.520
Baseline NIHSS score	15.30 ± 7.05	1.18 (1.12, 1.25)	< 0.001
ASPECTS	8.02 ± 1.45	0.90 (0.72, 1.12)	0.357
Glucose at admission	7.76 ± 2.85	1.13 (1.02,1.25)	0.017
Bridging intravenous thrombolysis	24 (10.13)	2.63 (1.04, 6.64)	0.040
Favorable collateral circulation	48 (20.25)	0.36 (0.12, 1.07)	0.066
Cardioembolic stroke	141 (59.49)	2.18 (1.04, 4.58)	0.040
Internal carotid artery occlusion	92 (38.82)	1.96 (1.00, 3.85)	0.049
Pneumonia during hospitalization	98 (41.35)	2.18 (1.11, 4.29)	0.024
Urinary tract infection during hospitalization	8 (3.38)	2.92 (0.67, 12.74)	0.153
Dialysis during hospitalization	1 (0.70)	Inf.(0.00, Inf)	0.991
Anticoagulation during hospitalization	64 (27.00)	0.17 (0.05, 0.57)	0.004
Use of invasive mechanical ventilation during hospitalization	51 (21.52)	17.54 (7.96, 38.67)	< 0.001
Use of vasopressors during hospitalization	32 (13.50)	13.86 (5.98, 32.16)	< 0.001
Sepsis during hospitalization	6 (2.53)	10.16 (1.80, 57.45)	0.001
Any intracerebral hemorrhage during hospitalization	102 (43.04)	4.92 (2.33, 10.39)	< 0.001
Symptomatic intracerebral hemorrhage during hospitalization	19 (8.02)	26.53 (8.20, 85.84)	< 0.001
NLR			
At admission	4.43 ± 3.71	1.08 (1.00, 1.17)	0.0578
Immediately after EVT	8.59 ± 5.41	1.05 (0.99, 1.11)	0.111
Within 12–24 h after EVT	10.03 ± 6.12	1.14 (1.07, 1.20)	< 0.001
Within 24–48 h after EVT	9.32 ± 5.27	1.13 (1.05, 1.22)	0.002
Within 48–72 h after EVT	10.09 ± 5.93	1.22 (1.12, 1.33)	< 0.001

*NLR* Neutrophil-to-lymphocyte ratio, *ASPECTS* Alberta Stroke Program Early CT Score, *BP* Blood pressure, *OR* Odds ratio, *CI* Confidence interval, *EVT* Endovascular treatment, *NIHSS* National Institute of Health Stroke Scale

^a^Data are n (%) or mean ± SD

After adjustments were made for confounding factors, multivariate analysis demonstrated that NLR obtained within 12 to 24 hours after EVT (odds ratio [OR] = 1.18; 95% confidence interval [CI]: 1.04, 1.33; *P* = 0.008), within 24 to 48 hours after EVT (OR = 1.16; 95% CI: 1.01, 1.35; *P* = 0.044), and within 48 to 72 hours after EVT (OR = 1.23; 95% CI: 1.03, 1.47; *P* = 0.021) were independently associated with mortality at 1 month after treatment (Table 3).

**Table 3:** Multivariate analysis of dynamic NLR using different models

NLR	Mortality at 1 mo					
	Crude model^**a**^		Adjusted model I^**b**^		Adjusted model II^**c**^	
	OR (95% CI)	***P*** value	OR (95% CI)	***P*** value	OR (95% CI)	***P*** value
At admission	1.08 (1.00, 1.17)	0.058	1.08 (0.99, 1.17)	0.087	1.00 (0.86, 1.16)	0.971
Immediately after EVT	1.05 (0.99, 1.11)	0.111	1.04 (0.98, 1.10)	0.219	1.05 (0.94, 1.17)	0.422
Within 12–24 h after EVT	1.14 (1.07, 1.20)	< 0.001	1.14 (1.07, 1.22)	< 0.001	1.18 (1.04, 1.33)	0.008
Within 24–48 h after EVT	1.13 (1.05, 1.22)	0.002	1.12 (1.03, 1.21)	0.005	1.16 (1.01, 1.35)	0.044
Within 48–72 h after EVT	1.22 (1.12, 1.33)	0.001	1.23 (1.12, 1.35)	< 0.001	1.23 (1.03, 1.47)	0.021

*NLR* Neutrophil-to-lymphocyte ratio, *OR* Odds ratio, *CI* Confidence interval, *EVT* Endovascular treatment

^a^Crude model: not adjusted for other covariates

^b^Adjusted model I: adjusted for sex and age

^c^Adjusted model II: sex; age; history of diabetes; history of chronic kidney disease; baseline NIHSS score; bridging intravenous thrombolysis; cardioembolic stroke; internal carotid artery occlusion; favorable collateral circulation; pneumonia during hospitalization; symptomatic intracerebral hemorrhage during hospitalization; sepsis during hospitalization; anticoagulation during hospitalization; use of invasive mechanical ventilation during hospitalization; use of vasopressors during hospitalization

**Fig. 2:**
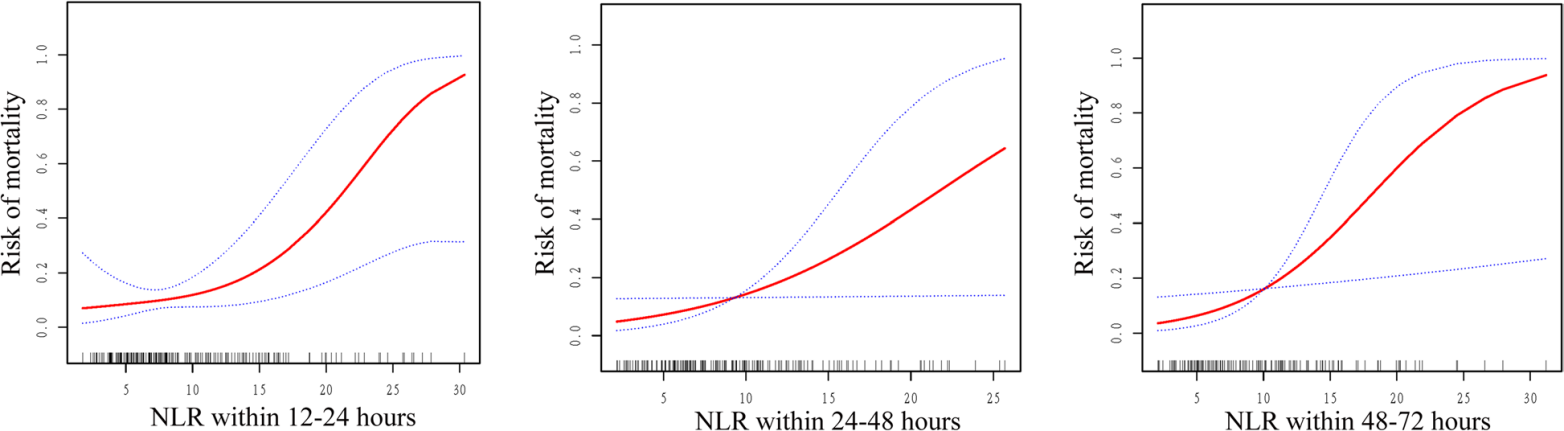
A nearly linear association between neutrophil-to-lymphocyte ratio (NLR) and mortality at 1 month after endovascular treatment (EVT) can be seen in a generalized additive model. A smooth curve fitting graph illustrates daily NLR in 237 patients (195 survivors, 42 nonsurvivors) for 3 consecutive days after EVT. The solid line and the dashed lines show the estimated value and its corresponding 95% confidence interval, respectively. Adjustments were made for sex; age; history of diabetes; history of chronic kidney disease; baseline NIHSS score; bridging intravenous thrombolysis; cardioembolic stroke; internal carotid artery occlusion; favorable collateral circulation; pneumonia during hospitalization; symptomatic intracerebral hemorrhage during hospitalization; sepsis during hospitalization; anticoagulation during hospitalization; use of invasive mechanical ventilation during hospitalization; use of vasopressors during hospitalization

**Fig. 3:**
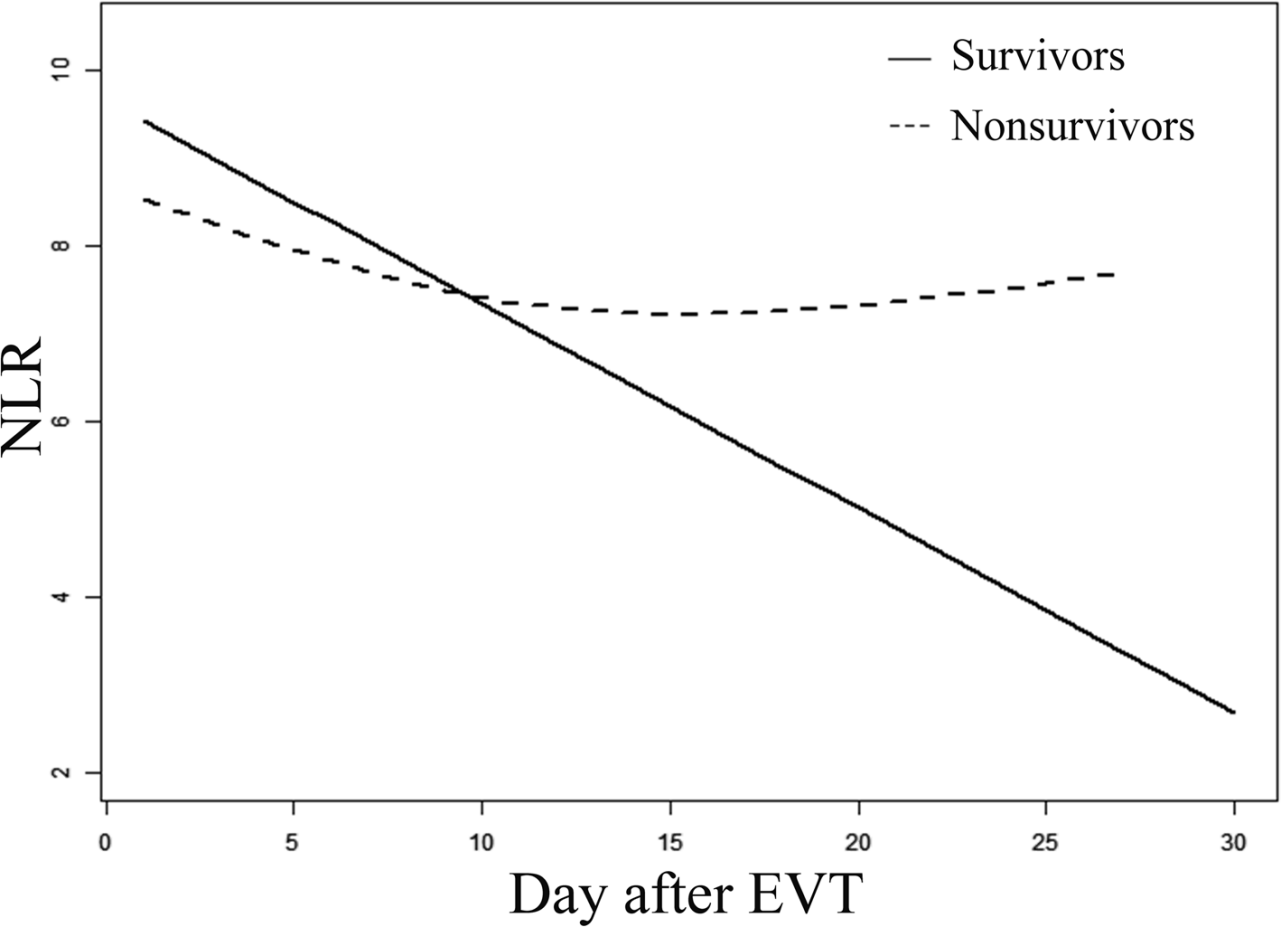
A nonlinear association between change in neutrophil-to-lymphocyte ratio (NLR) and time can be seen in a generalized additive model. A smooth curve fitting graph illustrates NLR in 237 patients (195 survivors, 42 nonsurvivors) based on the number of days since endovascular treatment (EVT). The solid line represents survivors; the dashed line represents nonsurvivors. Adjustments were made for sex; age; history of diabetes; history of chronic kidney disease; baseline NIHSS score; bridging intravenous thrombolysis; cardioembolic stroke; internal carotid artery occlusion; favorable collateral circulation; pneumonia during hospitalization; symptomatic intracerebral hemorrhage during hospitalization; sepsis during hospitalization; anticoagulation during hospitalization; use of invasive mechanical ventilation during hospitalization; use of vasopressors during hospitalization

There was a nearly linear relationship between NLR and mortality for 3 consecutive days after EVT (Fig. 2). We also found that there was a trend for NLR to decrease gradually over time; however, the NLR in survivors decreased significantly faster than in nonsurvivors (Fig. 3). The NLR over time differed between the 2 groups, and within 1 month after EVT, NLR in survivors had decreased by an average of 0.29 daily than in nonsurvivors (Table 4). After adjustments were made for multiple variables, this difference of 0.29 remained.

**Table 4:** Relationship between dynamic NLR and mortality in 237 patients at 1 month using a generalized additive mixed model

Outcome	Model I^**a**^		Model II^**b**^	
	β (95% CI)	***P*** value	β (95% CI)	***P*** value
Intercept^c^	9.086 (8.499, 9.673)	< 0.001	4.381 (1.230, 7.559)	0.007
Day^d^	−0.237 (−0.272, − 0.202)	< 0.001	−0.244 (− 0.279, − 0.208)	< 0.001
Death^e^	2.850 (1.391, 4.301)	< 0.001	1.243 (− 0.708, 3.1940)	0.213
Day × death^f^	0.288 (0.182, 0.394)	< 0.001	0.293 (0.186, 0.400)	< 0.001

*CI* Confidence interval, *NLR* Neutrophil-to-lymphocyte ratio

^a^Model I: not adjusted for other covariates

^b^Model II: adjusted for sex; age; history of diabetes; history of chronic kidney disease; baseline NIHSS score; bridging intravenous thrombolysis; cardioembolic stroke; internal carotid artery occlusion; favorable collateral circulation; pneumonia during hospitalization; symptomatic intracerebral hemorrhage during hospitalization; sepsis during hospitalization; anticoagulation during hospitalization; use of invasive mechanical ventilation during hospitalization; use of vasopressors during hospitalization

^c^Intercept: the mean NLR at day = 0 and death = 0

^d^Day: the mean decrease in NLR at death = 0 over time (daily)

^e^Death: the difference in NLR at day = 0 between nonsurvivors = 1 and survivors = 0

^f^Day × death: the mean decrease in NLR daily under the condition of nonsurvivors = 1 and survivors = 0

## Discussion

In this study, we assessed dynamic NLR in patients who underwent EVT for ischemic stroke with acute large artery occlusion and found that increased NLR in the early stage within 3 days after treatment was associated with an increased risk of mortality at 1 month. Within 1 month after EVT, the NLR of survivors had decreased more than that of nonsurvivors, even after adjustments were made for confounders. These results suggest that increased NLR over time are associated with a higher risk of mortality. This study further expands the impact of dynamic NLR on mortality in stroke patients.

Inflammation plays an important role in the process of ischemic stroke initiation, progression, and recovery [30]. In the acute stage of stroke, blood flow stagnation at the distal end of the thrombus may cause the arterial endothelium to release adhesion molecules, cytokines, and chemokines and to recruit white blood cells, mainly neutrophils, by occluding the distal cortical pial collateral vessels [5]. These white blood cells collect in ischemic cerebral vessels. Neutrophils in the blood enter ischemic or infarcted tissue through the damaged blood-brain barrier and transmit inflammatory factors. The local release of oxygen free radicals and matrix metalloproteinase-9 and inflammatory mediators such as cytokines (eg, interleukin-1β, TNF, and interleukin-6) further increases the risk of blood-brain barrier injury and reperfusion injury, malignant edema, and/or hemorrhagic transformation [7, 8]. However, the ability of T lymphocytes to bind platelets through P-selectin may play a key role in preventing hemorrhagic transformation after acute ischemic stroke [9, 10]. In the subacute phase of acute ischemic stroke (> 48 to 72 h), cerebral ischemic tissue may release cytokines and neurotransmitters and activate immune regulation systems such as the hypothalamus-pituitary axis and sympathetic nervous system, which can lead to down-regulation of systemic immune function and stroke-induced immunosuppression) [31, 32]. This in turn leads to a decrease in lymphocytes (especially T cells and natural killer cells), systemic immunosuppression, and an increased risk of infection [30]. Acute ischemic stroke causes distal organ damage and is associated with a high risk of non-neurological complications, including respiratory failure, cardiovascular dysfunction, liver and kidney damage, and altered immune and endocrine function [33]. These complications can affect prognosis and may have serious short-term and long-term adverse consequences. Thus, assessing the state of inflammation in the early poststroke period may provide clinicians with additional information about potential outcomes.

Recent studies have shown that NLR is associated with the prognosis of hemorrhagic stroke and increased cerebral hemorrhage [34–37]. Inflammation plays an important role in the mechanism of brain injury in hemorrhagic stroke. Therefore, by releasing cytotoxic mediators, increasing capillary permeability, and promoting disruption of the blood-brain barrier, neuro-inflammation plays a pivotal part in hematoma enlargement, brain cell death, brain edema formation and intracranial hypertension [38]. In the early stage of stroke, most of the white blood cells entering the brain come from peripheral blood, with neutrophils being the first hematopoietic cells to be recruited. Hence similar action mechanisms of inflammation in ischemic stroke and hemorrhagic stroke.

Previous studies have demonstrated a relationship between mortality and NLR at admission (OR = 1.19; 95% CI 1.03, 1.36; *P* = 0.02) [39]. Higher NLR (6.3 (IQR 4.4–10.8) vs 4.9 (IQR 3.1–7.4); *P* = 0.002) and greater temporal change in NLR (2.4 (IQR 1.1–7.6) vs 1.3 (IQR 0.1–3.7); *P* = 0.05) within 3 to 7 days after EVT were also associated with mortality at 90 days [40]. In the present study, we found that baseline NLR were not associated with mortality at 1 month (OR = 1.00; 95% CI: 0.86, 1.16; *P* = 0.971), but NLR obtained within 12 to 24 hours after EVT were associated with mortality at 1 month (OR = 1.18; 95% CI: 1.04, 1.33; *P* = 0.008). For every unit increase in NLR in the early stage, the risk of mortality increased by approximately 18%. Differences in findings between our study and previous research may be partially explained by differences in the enrolled populations (eg, more serious condition in patients requiring thrombectomy) and the stress reaction associated with undergoing EVT.

Our previous research has shown that increased NLR immediately after EVT is associated with an unfavorable prognosis (modified Rankin Scale score of 3–6) at 3 months for anterior circulation large vessel occlusion stroke (OR = 1.19; 95% CI: 1.07, 1.32; *P* = 0.002) [41], but in this study we did not find that increased NLR immediately after EVT was associated with death at 1 month(OR = 1.05; 95% CI: 0.94, 1.17; *P* = 0.422). Additionally, we found that NLR in patients still alive at 1 month decreased by an average of 0.29 daily than in nonsurvivors. To our knowledge, this is the first study to examine the association between persistent dynamic changes in NLR during hospitalization and mortality, thus extending previous research in several ways. First, we demonstrated the trends in dynamic NLR over time during hospitalization after successful revascularization. Second, we compared these trends in survivors and nonsurvivors to assess the association between dynamic NLR and mortality at 1 month after EVT. The NLR might be a biological marker of prognosis in patients undergoing EVT and could also sensitively reflect changes in the severity of a patient’s condition [12, 42]. Additionally, we found that the change in NLR over time was more informative regarding the risk of mortality than any individual NLR. Increased NLR over time should therefore prompt closer scrutiny of a patient’s condition. Because it is easy and inexpensive to obtain routine bloodwork results, performing this testing daily may help to elucidate disease progression and may assist clinicians with formulating more effective treatment measures. NLR-like inflammatory biomarkers, such as the systemic inflammatory response index (SIRI), have been shown to predict the prognosis of stroke patients who receive endovascular treatment and are successful recanalized [43]. However, even with complete or near complete recanalization, a higher admission SIRI increased the risk of a poor outcome at 3 months. Taken together, the advantage of these inflammatory markers is that they are easily available and accessible and cost-effective since they come from widely available laboratory variables that are typically collected in routine medical practices.

Our study had some limitations. First, this study was limited to patients who had experienced an anterior circulation stroke, and so the results cannot be extrapolated to the posterior circulation stroke population. Second, in order to study the mechanism of ischemia reperfusion injury, the enrolled patients had all undergone successful recanalization after thrombectomy; patients in whom revascularization was not successful were not included in the analysis. Third, some repeated measurements of NLR were missing for study patients. However, we used a generalized additive model and a generalized additive mixed model to analyze all collected data, and these models have been found to be useful when some data are missing. Finally, although we adjusted for multiple confounding factors, other potential confounding factors may have been missed in these analyses.

## Conclusion

We demonstrated the pathophysiological changes of dynamic NLR on stroke and the persistent effect of inflammation on ischemia-reperfusion injury. Because NLR can be easily obtained repeatedly, these measurements can be used to assess the changes in disease progress over time. These results suggest that physicians should be aware of the potential harm associated with persistently high NLR in patients who have undergone successful revascularization for anterior circulation large vessel occlusion stroke.

## Data Availability

The datasets used and/or analysed during the current study are available from the corresponding author on reasonable request.

## Abbreviations

NLR: Neutrophil-to-lymphocyte ratio
EVT: Endovascular treatment
CI: Confidence interval
OR: Odds ratio
CT: Tomography
DSA: Digital subtraction angiography
NIHSS: Health Stroke Scale
ASPECTS: Alberta Stroke Program Early CT Score
IQR: Interquartile range
